# Calcium-ligand variants of the myocilin olfactomedin propeller selected from invertebrate phyla reveal cross-talk with N-terminal blade and surface helices

**DOI:** 10.1107/S205979831901074X

**Published:** 2019-08-22

**Authors:** Shannon E. Hill, Hayeon Cho, Priyam Raut, Raquel L. Lieberman

**Affiliations:** a School of Chemistry and Biochemistry, Georgia Institute of Technology, 901 Atlantic Drive NW, Atlanta, GA 30332-0400, USA; b School of Biological Sciences, Georgia Institute of Technology, 310 Ferst Drive NW, Atlanta, GA 30318, USA

**Keywords:** olfactomedins, helix unwinding, calcium ions, propeller

## Abstract

Olfactomedins are a family of modular proteins found in multicellular organisms that all contain five-bladed β-propeller olfactomedin (OLF) domains. In support of differential functions for the OLF propeller, the available crystal structures reveal that only some OLF domains harbor an internal calcium-binding site with ligands derived from a triad of residues. For the myocilin OLF domain (myoc-OLF), ablation of the ion-binding site (triad Asp, Asn, Asp) by altering the coordinating residues affects the stability and overall structure, in one case leading to misfolding and glaucoma. Bioinformatics analysis reveals a variety of triads with possible ion-binding characteristics lurking in OLF domains in invertebrate chordates such as Arthropoda (Asp–Glu–Ser), Nematoda (Asp–Asp–His) and Echinodermata (Asp–Glu–Lys). To test ion binding and to extend the observed connection between ion binding and distal structural rearrangements, consensus triads from these phyla were installed in the myoc-OLF. All three protein variants exhibit wild-type-like or better stability, but their calcium-binding properties differ, concomitant with new structural deviations from wild-type myoc-OLF. Taken together, the results indicate that calcium binding is not intrinsically destabilizing to myoc-OLF or required to observe a well ordered side helix, and that ion binding is a differential feature that may underlie the largely elusive biological function of OLF propellers.

## Abstract 2

By installing calcium-ligand triads found in olfactomedin sequences from invertebrates, insight was gained into calcium-binding features across the protein family and new surface conformations were observed that may be relevant to elusive protein–protein interactions.

## 1. Introduction   

Olfactomedins (OLFs), modular proteins containing an OLF domain that are predominantly expressed in neural tissues, are involved in fundamental processes of cell growth, cell signaling and cell adhesion. Several members of the OLF protein family are further implicated in human diseases such as glaucoma, childhood obesity, and gastric and colorectal cancer (Anholt, 2014 ▸). Phylogenetically, the OLF family is divided into seven (Zeng *et al.*, 2005 ▸) or nine (Li *et al.*, 2019 ▸) subfamilies, based on sequence analysis, domain organization, tissue-expression pattern and implicated biological function.

**Figure 1:**
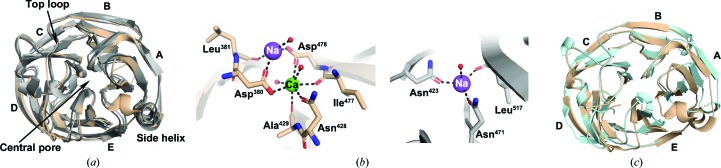
Myoc-OLF structures and comparison with other olfactomedin domains. (*a*) Superposition of myoc-OLF (beige; PDB entry 4wxs; Donegan *et al.*, 2015 ▸) with human olfactomedin-1 (grayscale; PDB entry 4xat; r.m.s.d = 0.62 Å; Hill *et al.*, 2015 ▸), human latrophilin 3 (PDB entry 5afb; r.m.s.d. 0.82 Å; Jackson *et al.*, 2016 ▸) and rat gliomedin (PDB entry 4xav; r.m.s.d. = 1.06 Å; Hill *et al.*, 2015 ▸). (*b*) Metal-ion centers in myoc-OLF (left) and rat gliomedin (right). (*c*) Superposition of myoc-OLF (PDB entry 4wxs) and myoc-OLF^D478S^ (light blue; PDB entry 6ou3).

High-resolution crystal structures of the ∼30 kDa five-bladed β-propeller OLF domain are available for four subfamily members: olfactomedin-1 (Pronker *et al.*, 2015 ▸; Hill *et al.*, 2015 ▸), latrophilin (Lu *et al.*, 2015 ▸; Ranaivoson *et al.*, 2015 ▸; Jackson *et al.*, 2016 ▸), myocilin (Donegan *et al.*, 2015 ▸) and gliomedin (Han & Kursula, 2015 ▸; Hill *et al.*, 2015 ▸). While the folds are nearly indistinguishable [Fig. 1 ▸(*a*)], one difference is the presence or absence of a heptacoordinate calcium ion bound in the central internal solvent-filled cavity of the β-propeller [Fig. 1 ▸(*b*)]. For the wild-type myocilin OLF domain (myoc-OLF^WT^), the calcium-binding residues include Asp380, Asn428 and Asp478, with additional backbone carbonyls and water molecules completing the coordination environment [Fig. 1 ▸(*b*)]. Changes to the calcium coordination in myoc-OLF^WT^ have a significant impact on the stability and overall structure: mutations of Asp380, a site with glaucoma-causing mutations, decrease the thermostability of myoc-OLF (Donegan *et al.*, 2012 ▸; Hill *et al.*, 2019 ▸), while mutations of Asp478 are stabilizing (Hill *et al.*, 2019 ▸). Paradoxically, Asp478 variants adopt non-native structures, with significant loop disorder and structural rearrangements in the N-terminal propeller blade A [Fig. 1 ▸(*c*)] (Hill *et al.*, 2019 ▸). Given that evolutionary adaptation does not favor overall protein stabil­ity (DePristo *et al.*, 2005 ▸; Tokuriki *et al.*, 2008 ▸; Tokuriki & Tawfik, 2009 ▸), these results suggest there is a functional reason that some OLF domains possess a binding site for calcium and some do not. Such inferences are valuable because the explicit biological function of most OLF domains remains largely enigmatic (Anholt, 2014 ▸), and it is not yet known to what extent the binding partners and function of different OLFs might be similar.

Here, we first used sequence alignments to tabulate the residues found at the site of the calcium-binding triad in myoc-OLF across the olfactomedin family, focusing on invertebrates for which OLF domains have not been investigated previously. After installing selected common triad residues into myoc-OLF, we evaluated the protein stability and stabilization in the presence of calcium. Crystal structures of these variants further elaborate on the metal-binding properties in the olfactomedin family as well as revealing motions and features of myoc-OLF that have not been observed previously. Our results clarify that calcium binding is not intrinsically destabilizing to myoc-OLF and support the hypothesis that differential metal ion-binding properties are functionally relevant across the broader protein family.

## 2. Materials and methods   

### 2.1. Phylogenetic analysis   

OLF-domain sequences curated in the SMART database (Letunic *et al.*, 2015 ▸) for Arthropoda (219 sequences), Echino­dermata (17 sequences) and Nematoda (130 sequences) were downloaded. The sequence corresponding to our myoc-OLF construct was added manually to each set prior to alignment with *MUSCLE* on the EMBL server (Madeira *et al.*, 2019 ▸). The residues aligning with triad positions 380, 428 and 478 of human OLF were extracted and either plotted using *GraphPad Prism* or visualized with *WebLogo* (Crooks *et al.*, 2004 ▸). Extracted sequences and full alignments will be supplied on request to the corresponding author.

### 2.2. Molecular biology   

Site-directed mutagenesis (SDM) was performed on one of two previously published myoc-OLF^WT^ plasmids, which contain myoc-OLF (residues 228–504) fused to maltose-binding protein and either an intervening factor Xa (Burns *et al.*, 2010 ▸; for myoc-OLF^N428E/D478S^) or Tobacco etch virus (TEV; Hill *et al.*, 2014 ▸; for myoc-OLF^N428E/D478K^ and myoc-OLF^N428D/D478H^) protease cleavage site. Primers (Supplementary Table S1) were designed using the *PrimerX* software and were purchased from MWG Operon. SDM was conducted by following the protocol accompanying the Agilent QuikChange Lightning kit. The fidelity of the plasmids was verified by DNA sequencing (GenScript).

### 2.3. Expression and purification of myoc-OLF variants   

As described previously (Hill *et al.*, 2014 ▸), the plasmids were transformed into Rosetta-gami 2 competent cells and grown on LB agar plates supplemented with 60 µg ml^−1^ ampicillin (AMP) and 34 µg ml^−1^ chloramphenicol (CAM). A single colony was used to inoculate 250 ml Luria broth containing selective AMP/CAM antibiotics and was incubated overnight at 37°C with shaking (225–250 rev min^−1^). The starter culture (25 ml) was then used to re-inoculate 1 l Superior Broth followed by shaking at 37°C until the cell suspension reached an optical density (OD) of ∼1.5–1.7 at 600 nm. At this point, the temperature was reduced to 18°C, the flasks were equilibrated for 1.5 h at 18°C and protein expression was induced with 500 µ*M* isopropyl β-d-1-thiogalactopyranoside (IPTG). The cells were harvested by centrifugation the following day, flash-frozen in liquid nitrogen and stored at −80°C. The cells were solubilized in amylose wash buffer (10 m*M* Na_2_HPO_4_, 10 m*M* KH_2_PO_4_, 200 m*M* NaCl, 1 m*M* EDTA) with 1× cOmplete protease-inhibitor cocktail (Roche), lysed twice using a French press and ultracentrifuged at 110 000*g* to remove cellular debris. The soluble fraction was loaded onto an amylose affinity column equilibrated with amylose wash buffer. Fractions eluting from the amylose affinity column were further fractionated on a Superdex 75 size-exclusion column equilibrated with gel-filtration buffer (10 m*M* Na_2_HPO_4_, 10 m*M* KH_2_PO_4_, 200 m*M* NaCl) to isolate monomeric MBP-myoc-OLF. For thermal stability measurements and crystallographic studies, myoc-OLF variants were cleaved from MBP by factor Xa or TEV protease. MBP was removed from cleaved myoc-OLF variants by amylose affinity chromatography followed by Superdex 75 size-exclusion chromatography in gel-filtration buffer. Protein purity was assessed by 12 or 15% SDS–PAGE analysis with Coomassie staining.

### 2.4. Thermal stability and effect of Ca^2+^   

Thermal stability in the presence and absence of calcium was evaluated by differential scanning fluorimetry (DSF) as described previously (Donegan *et al.*, 2012 ▸). Briefly, an Applied Biosciences Step-One Plus Real-Time PCR instrument was utilized to conduct a thermal melt from 25 to 95°C with a 1°C min^−1^ increase in the presence of SYPRO Orange (Invitrogen) either in the absence or the presence of 10 m*M* CaCl_2_. The melting temperature (*T*_m_) was then calculated as the midpoint of unfolding using a Boltzmann sigmoid equation using the *Igor* software. Evidence for ion binding was assessed as an increase in *T*_m_ of greater than 2.5°C in the presence of 10 m*M* metal ion.

### 2.5. Crystallization and structure determination   

Prior to crystallization, the proteins were exchanged into 10 m*M* HEPES pH 7.5 and concentrated to 12 mg ml^−1^ for myoc-OLF^N428E/D478K^, 22 mg ml^−1^ for myoc-OLF^N428D/D478H^ and 15 mg ml^−1^ for myoc-OLF^N428E/D478S^ using Amicon filtration devices with a molecular-weight cutoff of 10 kDa. For myoc-OLF^N428E/D478K^ the reservoir solution (1 ml) was composed of 20% PEG 3350, 0.2 *M* bis-Tris pH 6, 0.2 *M* magnesium formate. Hanging drops (2 µl) were prepared on a silanized glass cover slip (Hampton Research) by mixing the concentrated myoc-OLF^N428E/D478K^ with the reservoir solution, and crystals formed after ∼3 weeks of equilibration at 16°C. Crystals of myoc-OLF^N428E/D478K^ were harvested and cryoprotected for synchrotron data collection using the reservoir solution supplemented with 20% glycerol. Crystals of myoc-OLF^N428D/D478H^ were grown using a reservoir solution composed of 10% PEG 8000, 50 m*M* MgCl_2_ (500 µl), where the PEG 8000 solution had been passed through a 0.2 µm filter. The hanging-drop method was again used with glass cover slips but with a 4 µl droplet and an equal ratio of protein and reservoir solutions. Crystals appeared after equilibration for ∼3 weeks at 16°C and were harvested and cryoprotected with reservoir solution supplemented with 25% glycerol. For myoc-OLF^N428E/D478S^, the conditions were similar to those for myoc-OLF^N428D/D478H^ except that the reservoir was composed of 20% PEG 8000 that had not been filtered and 100 m*M* MgCl_2_ (1 ml), and the cryoprotectant was composed of reservoir solution supplemented with 20% glycerol. Diffraction data were collected on the Southeast Regional Collaborative Access Team (SER-CAT) 22-ID beamline and were processed using *HKL*-2000 (Minor *et al.*, 2006 ▸). Molecular replacement was performed with *Phaser* (McCoy *et al.*, 2007 ▸) using the myoc-OLF^D478N^ (PDB entry 6ou2; Hill *et al.*, 2019 ▸) polypeptide chain as the search model after the removal of all noncovalently bonded small molecules. *Coot* (Emsley *et al.*, 2010 ▸) and *phenix.refine* (Afonine *et al.*, 2012 ▸) were used to iteratively build and refine the model. For the anomalous data set for myoc-OLF^N428E/D478S^, data were collected at SER-CAT and were processed with *XDS* (Kabsch, 2010 ▸). One monomer from the myoc-OLF^N428E/D478S^ high-resolution data set was used as the search model for molecular replacement in *Phaser* and anomalous maps were generated after one round of refinement. *Coot* (Emsley *et al.*, 2010 ▸) and *PyMOL* (v.1.8; Schrödinger) were used for structural alignments. Metal-coordination properties were analyzed using the *CheckMyMetal* server (Zheng *et al.*, 2017 ▸). Figures were generated in *PyMOL* or *Coot*.

## 3. Results and discussion   

### 3.1. Phylogenetic analysis   

**Figure 2:**
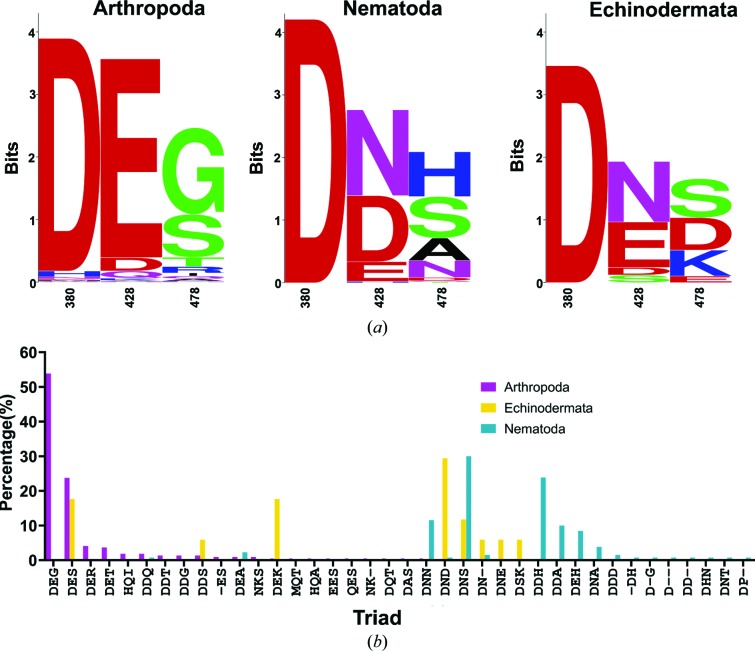
Analysis of invertebrate olfactomedin sequences. (*a*) *WebLogo* images depicting the sequence variations at calcium-binding residues in myoc-OLF. (*b*) Identified triad sequences across the three inspected phyla.

Sequences of OLF-like proteins from three invertebrate phyla (Arthropoda, Nematoda and Echinodermata) were downloaded from the SMART database (Letunic *et al.*, 2015 ▸) (accession no. SM00284). Alignment of sequences from these phyla together with myoc-OLF^WT^ using *MUSCLE* (Edgar, 2004*a* ▸,*b* ▸) revealed intriguing sequence permutations within the internal cavity (Fig. 2 ▸). The position corresponding to Asp380 is highly conserved across the species inspected [Fig. 2 ▸(*a*)], with the exception of a very few Arthropoda (13/219) that appear to have His (5/219), Asn (3/219), Gln (1/219), Met (1/219) or Glu (1/219) at this position [Supplementary Fig. S1(*a*)]; two sequences have no residue aligned at this position (2/219), but this may be a consequence of the sequence quality. By contrast, the other two positions are more variable across the three inspected phyla [Fig. 2 ▸(*a*)]. The position corresponding to Asn428 in myoc-OLF^WT^ [Fig. 2 ▸(*a*), Supplementary Fig. S1(*a*)] is dominated by Glu (88%) in Arthropoda, whereas in Nematoda Asn, Asp or Glu are common and in Echinodermata Asn, Asp, Glu or Ser are present. For the position corresponding to Asp478 in myoc-OLF^WT^ [Fig. 2 ▸(*a*), Supplementary Fig. S1(*a*)], Arthropoda most often have Ser and Gly but numerous other polar residues can be found, whereas for Nematoda His, Ser, Ala and Asn dominate and for Echinodermata Ser, Asp and Lys are most common.

The triads of available sequences further predict differential calcium ion-binding properties. The most common calcium-ion ligands among crystal structures in the Protein Data Bank (PDB) include the negatively charged side chains Glu and Asp, followed by Asn (Lu *et al.*, 2012 ▸). Six triads appear most frequently in OLF-like proteins, with different distributions across the three phyla. Arthropoda exhibits the highest apparent overall variability in triads as well as the highest total number of sequences available for analysis. In Arthropoda [Fig. 2 ▸(*b*)], Asp–Glu–Gly is the most prevalent triad (54%) and is unique among the inspected phyla. Asp–Glu–Ser is the second-ranked triad in Arthropoda. The most prevalent in Nematoda is Asp–Asn–Ser, which coincidentally corresponds to the triad in our previously characterized myoc-OLF^D478S^ variant that failed to bind calcium (Hill *et al.*, 2019 ▸). Asp–Asp–His is the second-ranked triad in Nematoda. For Echinodermata, the most prevalent triad is Asp–Asn–Asp, as seen in myoc-OLF^WT^, followed by Asp–Glu–Ser, which is second-ranked in Arthropoda, and Asp–Glu–Lys, which is seen in the sequence of sea urchin amassin (Hillier *et al.*, 2007 ▸). To survey the OLF triads for calcium binding and to test whether calcium binding can be reverted by simultaneously altering Asn428 to an acidic Asp or Glu, we replaced the triad Asp–Asn–Asp with Asp–Glu–Ser, Asp–Asp–His and Asp–Glu–Lys via site-directed mutagenesis of Asn428 and Asp478.

### 3.2. Thermal stability and calcium stabilization of myoc-OLF^N428E/D478S^, myoc-OLF^N428E/D478K^ and myoc-OLF^N428D/D478H^   

After mutagenesis, the expression and purification of the calcium-triad variants proceeded as for the previously generated myoc-OLF^WT^, and the thermal stabilities of purified variants eluting as monomers (Supplementary Fig. S2) were measured by DSF in the absence and presence of calcium (Table 1 ▸). In the case of myoc-OLF^N428E/D478S^ the conversion of Asn428 to Glu decreases the stability compared with the previously characterized myoc-OLF^D478S^, reverting to the value for myoc-OLF^WT^. Addition of calcium to the reaction used for DSF leads to an 8.8°C increase in the melting temperature (*T*_m_), indicating that myoc-OLF^N428E/D478S^ can bind calcium, albeit utilizing a different ligand environment compared with myoc-OLF^WT^. For myoc-OLF^N428E/D478K^ and myoc-OLF^N428D/D478H^ the measured *T*_m_ values were similar to that for myoc-OLF^D478S^. For myoc-OLF^N428E/D478K^ the difference upon the addition of calcium is within the error of DSF (±2°C), suggesting that myoc-OLF^N428E/D478K^ cannot bind calcium; for myoc-OLF^N428D/D478H^ a marginal stabilization was observed with calcium, suggesting weak if any calcium binding.

### 3.3. Crystal structures of myoc-OLF^N428E/D478S^, myoc-OLF^N428E/D478K^ and myoc-OLF^N428D/D478H^   

**Figure 3:**
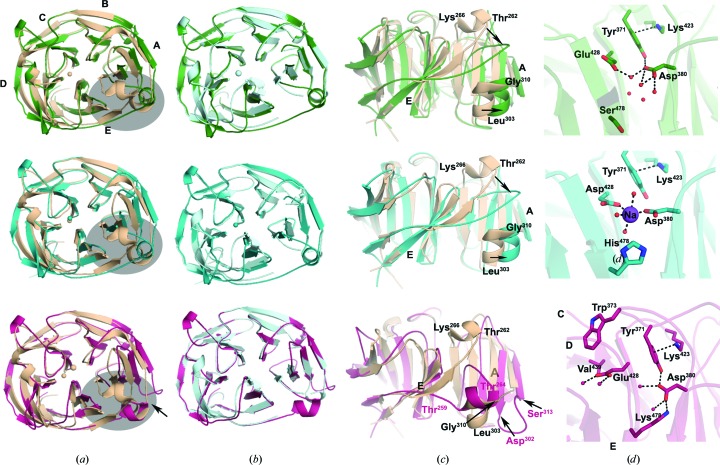
Structural comparison of myoc-OLF^N428E/D478S^ (green), myoc-OLF^N428D/D478H^ (teal) and myoc-OLF^N428E/D478K^ (magenta). (*a*) Superposition with myoc-OLF^WT^ (beige; PDB entry 4wxs), top face view. For myoc-OLF^N428E/D478S^ and myoc-OLF^N428D/D478H^, one monomer of the asymmetric unit is depicted. (*b*) Superposition with myoc-OLF^D478S^ (light blue; PDB entry 6ou3), top face view. (*c*) Enlargement of the blade E–A interface, showing movement of the side helix, in a side-on orientation approximately 90° from (*a*) and (*b*). (*d*) Enlargement of the triad in the central cavity. Dashed measurements are 2.0–2.5 Å except for the Tyr371–Lys423 cation–π interaction, which is ∼3.5 Å.

To clarify the structural changes accompanying the installed calcium-ligand triads, we next solved the crystal structures of each variant at ∼2 Å resolution (Tables 2 ▸ and 3 ▸). Based on the rearrangements that we observed upon ablating calcium binding in myoc-OLF^D478S^, we predicted that myoc-OLF^N428E/D478S^ would adopt a structure similar to that of myoc-OLF^WT^, and that myoc-OLF^N428E/D478K^ and myoc-OLF^N428D/D478H^ would appear more like myoc-OLF^D478S^. Overall, all three of our structures superimpose well with myoc-OLF^WT^ [r.m.s.d.s of ∼0.7–0.9 Å; Fig. 3 ▸(*a*)] but overlay better with myoc-OLF^D478S^ [r.m.s.d.s of ∼0.3–0.4 Å; Fig. 3 ▸(*b*)]. Myoc-OLF^N428E/D478S^ and myoc-OLF^N428D/D478H^ are most similar to each other (r.m.s.d. of 0.2 Å); either paired with myoc-OLF^N428E/D478K^ has an r.m.s.d. of ∼0.4 Å [Supplementary Fig. S3(*a*)]. However, as described below, these values belie nuances that reinforce the notion that the internal calcium-binding site affects surface features, and further implicate the helices and cleft between blades E and A as a likely contact surface for protein interactions in myoc-OLF.

In our structures of myoc-OLF^N428D/D478H^ [two monomers in the asymmetric unit; r.m.s.d. of 0.2 Å; Supplementary Fig. S3(*b*)] and myoc-OLF^N428E/D478S^ [two molecules in the asymmetric unit; r.m.s.d. of 0.2 Å; Supplementary Fig. S3(*c*)], the side helix (residues 303–310) is well ordered, as in myoc-OLF^WT^; however, the helix is shifted ∼3 Å closer to blade A [top and middle in Figs. 3(*b*) and 3(*c*) ▸], which is the configuration seen for myoc-OLF^D478S^ [top and middle in Fig. 3 ▸(*c*)]. Other similarities to myoc-OLF^D478S^ include disordered loops in monomer *A* (residues 291–292) and in monomer *B* (residues 266–267 and 291–293). For myoc-OLF^N428D/D478H^, nearly all residues were traced; residues 291–292 in monomer *A* and residues 292–293 in monomer *B* could not be traced, although several loops in both molecules were difficult to model, suggesting overall loop flexibility for this variant as well. Among the well ordered loops is one encompassing residues 259–268, which is shifted ∼5 Å in myoc-OLF^N428D/D478H^ and myoc-OLF^N428E/D478S^ compared with the analogous residues forming the top helical turn in myoc-OLF^WT^ [top and middle in Figs. 3 ▸(*a*) and 3 ▸(*c*)]. This shift is not propagated, as the loop connecting blade D to E adopts a similar conformation to those in myoc-OLF^WT^ and myoc-OLF^D478S^ [top and middle in Figs. 3 ▸(*a*) and 3 ▸(*b*)], and may be a crystallization artifact.

Unique to the myoc-OLF^N428D/D478H^ structure, we were able to trace the far N-terminal residues 235–244 of our myoc-OLF construct, which are part of the 60-amino-acid linker region that connects the N-terminal coiled coils of myocilin to its OLF domain, as well as the C-terminal residues 503–504 that complete the OLF domain [Supplementary Fig. S3(*d*)]. The main and side chains of Lys503 and the carboxy-terminus form several hydrogen-bonding interactions with residues 242–243. The far N-terminal region adopts an extended loop conformation, forming crystal contacts [Supplementary Fig. S3(*d*)] with (i) a side helix of one symmetry-related monomer *A*, (ii) the outermost strand of blade E in a second symmetry-related monomer *A* and (iii) two loops (439–446 and 468–475) of a symmetry-related monomer *B*.

In the vicinity of Asp380 in both myoc-OLF^N428E/D478S^ and myoc-OLF^N428D/D478H^ are two >8σ peaks in the difference Fourier (*F*_o_ −*F*_c_) electron-density map [Supplementary Figs. S4(*a*) and S4(*b*)]. Given the strong (for myoc-OLF^N428E/D478S^) and marginal (myoc-OLF^N428D/D478H^) stabilization by Ca^2+^ seen by DSF, we considered assigning either of these difference peaks as a Ca^2+^, but were ultimately unable to confirm calcium in either variant. Calcium coordination for the more likely variant myoc-OLF^N428E/D478S^ was not readily satisfied by the available protein ligands or water (not shown). To support the absence of Ca^2+^ in the structure, we employed a strategy used recently to identify Ca^2+^ ions by anomalous scattering (Hegde *et al.*, 2017 ▸). Our data only reveal anomalous difference peaks from sulfur [Cys245–Cys435 disulfide (not shown) and selected methionines; Supplementary Fig S4(*a*)]. As attempts to model cations from protein or crystallization buffer salts (Mg^2+^, Na^+^ and K^+^) were met with similarly ambiguous coordination environments, the final model for myoc-OLF^N428E/D478S^ contains waters clustered near Asp380 and Glu428 [Fig. 3 ▸(*d*), top]. For myoc-OLF^N428D/D478H^, the coordination environment is most consistent with a pentacoordinated metal ion, assigned in our model as Na^+^; consistent with DSF results, this metal ion does not appear to be a well ordered Ca^2+^, as poor thermal factors and/or occupancies result when this metal is replaced with Ca^2+^ (not shown). In sum, unlike myoc-OLF^WT^ (Donegan *et al.*, 2012 ▸), neither myoc-OLF^N428D/D478H^ nor myoc-OLF^N428E/D478S^ copurifies with a fully occupied Ca^2+^, but both the Nematoda triad Asp–Asp–His and the Arthropoda triad Asp–Glu–Ser appear to be able to support metal-ion binding, perhaps in certain bio­logical contexts.

The 1.5 Å resolution crystal structure of myoc-OLF^N428E/D478K^ reveals a striking swap of the two helix-containing loops in the cleft between blades E and A [Figs. 3 ▸(*a*) and 3 ▸(*b*), bottom]. Instead of not being visible (myoc-OLF^D478S^), or adopting a well ordered and helical loop at the top face (wild type), residues 260–265 are shifted ∼10 Å from the original top-face helical loop and are now in the approximate location of the myoc-OLF^WT^ side helix. In turn, the residues previously forming the side helix (303–310) form an extended loop conformation. Finally, unlike myoc-OLF^D478S^, the loop composed of residues 291–295 is ordered, although shifted ∼3 Å from its defined position in myoc-OLF^WT^. These shifts are not readily explained by crystal contacts, as myoc-OLF^N428E/D478K^ crystallized in the same space group and with the same unit-cell parameters as myoc-OLF^D478N^ (PDB entry 6ou2). In myoc-OLF^D478N^ residues 260–265 and 291–295 could not be traced and residues 303–310 form a WT-like side helix. In the central cavity, a salt bridge between Asp380 and Lys478 (2.6 Å) is observed, with the terminal N^∊^ atom occupying the position of the Na^+^ ion modeled in myoc-OLF^WT^. In addition, Lys478 is within hydrogen-bonding distance of the main-chain carbonyls of Gly326 and Leu381, and Asp380 forms a new hydrogen-bonding interaction with Tyr371 (2.7 Å). The position of Tyr371 appears to be shifted ∼0.6 Å compared with the wild type, but still forms a cation–π interaction with Lys423; both of these residues are conserved in Echinodermata OLFs with the Asp–Glu–Lys triad. Like Asn428 in myoc-OLF^D478S^, Glu428 is no longer in the central cavity and instead forms hydrogen bonds to water molecules present in a cavity between blades D and E. In myoc-OLF^WT^ this cavity is largely hydrophobic, and is lined with Trp373, Val439, Phe467 and Ile477. In the corresponding Echinodermata OLFs this pocket should be more polar, with Trp373 replaced with a Ser and Val439 replaced with a Thr. Overall, OLF domains harboring the Asp–Glu–Lys triad do not appear to be capable of metal-ion binding in the central pore.

It is widely accepted that protein flexibility is important in biomolecular recognition (Boehr *et al.*, 2009 ▸), and the consensus to date is that OLF domains, similar to many β-propellers, are likely to be involved in protein–protein interactions. Still, the currently available OLF-domain crystal structures show nearly indistinguishable folds across several family members and the binding partners for most olfactomedins are unknown, as are the triggers or structural changes that might be needed to accommodate such interactions. By altering the ion-binding residues in myoc-OLF to those found in invertebrate OLFs in this study, we learned about calcium-binding properties as well as gained access to new stable conformations of myoc-OLF. Calcium binding in myoc-OLF is not needed to observe the ordered side helix seen in Ca^2+^-loaded myoc-OLF^WT^, and the blade E–A interface appears to have an affinity for helices in at least three configurations. To date, the only other olfactomedin domain captured structurally in multiple configurations is latrophilin 3 (LPHN3), which harbors an Asn–Asp–Asp triad, which is coincidentally permuted in sequence compared with the myoc-OLF triad Asp–Asn–Asp. In the structure of mouse latrophilin 3 (*Mm*LPHN3; PDB entry 4rml; Ranaivoson *et al.*, 2015 ▸), the long top loop adopts an open conformation that is unique among all other OLF structures [Supplementary Fig. S5(*a*)] and an Mg^2+^ ion was found to be hexacoordinated to the equivalent of Asp478 [Asp436; Supplementary Fig. S5(*b*)]. In other LPHN3 structures, including the complex with the binding partner FLRT3, Ca^2+^ is bound with some slight variations in its coordination environment [for example in PDB entry 4rmk; Supplementary Fig. S5(*c*)], and this top loop is in the typical closed conformation [see Fig. 1 ▸(*a*)]. It is tempting to suggest that motion of the long loop is relevant to other OLFs, but we have not observed changed top-loop conformations in any structures of myoc-OLF variants to date. Our results seem to suggest that the blade A–E surface is both sensitive to the status of calcium binding in the central pore and may be an interface that is adaptable for interaction with a binding partner. In sum, although the details are likely to differ across olfactomedins, metal binding in OLF appears to trigger different conformations of loops on the OLF propeller. Such OLF calcium variants could be useful in experiments intended to decipher the binding partners and thus the functions of this large protein family.

## Supplementary Material

PDB reference: myocilin olfactomedin domain, N428D/D478H variant, 6pkd

PDB reference: N428E/D478S variant, 6pke

PDB reference: N428E/D478K variant, 6pkf

Supporting information for publication. DOI: 10.1107/S205979831901074X/mn5121sup1.pdf

## Figures and Tables

**Table 1:** Thermal stability measurements

Phylum	Protein	*T*_m_ (°C)	Stability comparison	Δ*T*_m_ (°C) with Ca^2+^	Ca^2+^ binding?
	Myoc-OLF^WT^	51.7 ± 0.3†		+7.2†	Yes
	Myoc-OLF^D478S^	58.7 ± 0.4†	Higher than WT	−1.1†	No
Arthropoda	Myoc-OLF^N428E/D478S^	53.4 ± 0.2	WT-like	+8.8	Yes
Echinodermata	Myoc-OLF^N428E/D478K^	58.7 ± 0.2	D478S-like	+1.4	No
Nematoda	Myoc-OLF^N428D/D478H^	57.2 ± 0.2	D478S-like	+2.7	Ambiguous

† Values for Myoc-OLF^WT^ are from Donegan *et al.* (2012 ▸); values for Myoc-OLF^D478S^ are from Hill *et al.* (2019 ▸).

**Table 2:** Crystallization

	Myoc-OLF^N428E/D478K^	Myoc-OLF^N428D/D478H^	Myoc-OLF^N428E/D478S^
Method	Hanging drop	Hanging drop	Hanging drop
Plate type	24-well	24-well	24-well
Temperature (°C)	16	16	16
Protein concentration (mg ml^−1^)	12	22	15
Buffer composition of protein solution	10 m*M* HEPES pH 7.5	10 m*M* HEPES pH 7.5	10 m*M* HEPES pH 7.5
Composition of reservoir solution	20% PEG 3350, 0.2 *M* bis-Tris pH 6, 0.2 *M* magnesium formate	10% PEG 8000, 50 m*M* MgCl_2_	20% PEG 8000, 100 m*M* MgCl_2_
Volume and ratio of drop	2 µl, 1:1	4 µl, 1:1	4 µl, 1:1
Volume of reservoir (ml)	1	0.5	1

**Table 3:** Data-collection and refinement statistics Values in parentheses are for the outer shell.

	Myoc-OLF^N428E/D478K^	Myoc-OLF^N428D/D478H^	Myoc-OLF^N428E/D478S^	Myoc-OLF^N428E/D478S^
PDB code	6pkf	6pkd	6pke	N/A (anomalous)
Wavelength (Å)	1.00	1.00	1.00	1.48
Resolution range	31.90–1.48 (1.54–1.48)	33.60–1.90 (1.97–1.90)	32.93–1.88 (1.95–1.88)	47.70–2.14 (2.22–2.14)
Space group	*P*2_1_2_1_2_1_	*P*12_1_1	*P*12_1_1	*P*12_1_1
*a*, *b*, *c* (Å)	45.51, 58.69, 89.51	48.55, 47.34, 97.08	48.22, 47.29, 97.02	48.23, 47.2, 50.15
α, β, γ (°)	90, 90, 90	90, 100.20, 90	90, 100.28, 90	90, 108.01, 90
Total reflections	446529 (41877)	137083 (11726)	108472 (9324)	323992 (31812)
Unique reflections	40126 (3773)	33852 (3266)	34569 (3330)	23125 (2286)
Multiplicity	11.1 (11.1)	4.0 (3.6)	3.1 (2.8)	14.0 (13.9)
Completeness (%)	99.28 (95.49)	98.06 (95.18)	97.60 (94.80)	99.73 (99.05)
Mean *I*/σ(*I*)	31.73 (7.78)	11.68 (3.88)	9.73 (2.54)	18.68 (3.64)
Wilson *B* factor (Å^2^)	11.92	19.27	25.49	33.29
*R*_meas_	0.129 (1.149)	0.166 (0.542)	0.139 (0.548)	0.0975 (0.559)
*R*_p.i.m._	0.0390 (0.351)	0.0806 (0.275)	0.0733 (0.307)	0.0258 (0.148)
CC_1/2_	0.998 (0.710)	0.99 (0.753)	0.993 (0.690)	0.999 (0.947)
CC*	1.000 (0.911)	0.997 (0.927)	0.998 (0.904)	1.000 (0.986)
Reflections used in refinement	40100 (3771)	33707 (3255)	34533 (3316)	23120 (2285)
Reflections used for *R*_free_	1999 (188)	2002 (194)	1992 (199)	2315 (231)
*R*_work_	0.151 (0.167)	0.183 (0.214)	0.176 (0.213)	0.227 (0.360)
*R*_free_	0.181 (0.196)	0.208 (0.239)	0.219 (0.250)	0.301 (0.395)
CC(work)	0.965 (0.946)	0.962 (0.851)	0.958 (0.892)	0.951 (0.772)
CC(free)	0.941 (0.902)	0.945 (0.803)	0.945 (0.837)	0.919 (0.668)
No. of non-H atoms				
Total	2387	4566	4399	2094
Macromolecules	2143	4226	4092	2038
Ligands	6	2	0	0
Solvent	234	338	307	56
No. of protein residues	259	526	512	256
R.m.s.d., bonds (Å)	0.024	0.008	0.007	0.013
R.m.s.d., angles (°)	2.17	0.89	0.92	1.61
Ramachandran statistics				
Favored (%)	96.11	96.14	95.42	90.87
Allowed (%)	3.89	3.28	4.38	8.33
Outliers (%)	0.00	0.58	0.20	0.79
Rotamer outliers (%)	0.43	0.44	0.23	0.45
Clashscore	9.87	9.24	7.22	16.75
Average *B* factors (Å^2^)				
Overall	19.48	24.63	32.13	44.78
Macromolecules	18.41	24.29	31.80	45.00
Ligands	27.50	44.69	N/A	N/A
Solvent	29.14	28.83	36.59	36.57
