# Supplementary material for: Calcium-ligand variants of the myocilin olfactomedin propeller selected from invertebrate phyla reveal cross-talk with N-terminal blade and surface helices
Source: Acta Crystallogr D Struct Biol. 2019 Aug 22;75(Pt 9):817–24. doi: 10.1107/S205979831901074X (PMC6719662; doi:10.1107/S205979831901074X)
Supplement: Supplementary file 1 [file d-75-00817-sup1.pdf]

STRUCTURAL BIOLOGY

Volume 75 (2019)

Supporting information for article:

Calcium-ligand variants of the myocilin olfactomedin propeller selected from invertebrate phyla reveal cross-talk with N-terminal blade and surface helices

Shannon E. Hill, Hayeon Cho, Priyam Raut and Raquel L. Lieberman

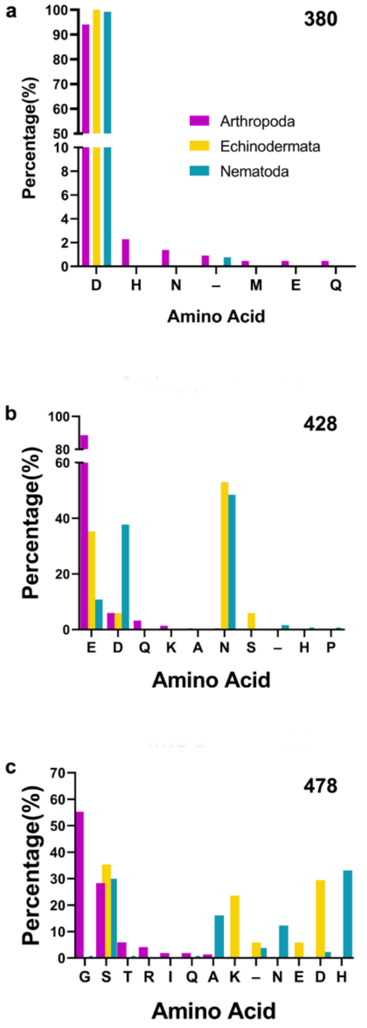

Figure S1 Frequency of residues at individual triad positions, (a) residue 380, (b) residue 428 and (c) residue 478, for each phylum inspected.

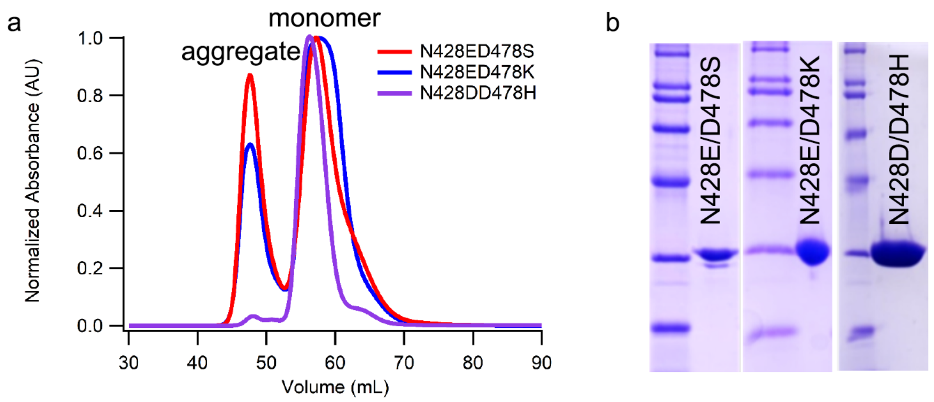

Figure S2 Protein purification. (a) Overlay of Superdex 75 size exclusion chromatogram of fusion proteins of each variant. (b) Final SDS-PAGE analysis of cleaved proteins used in this study.

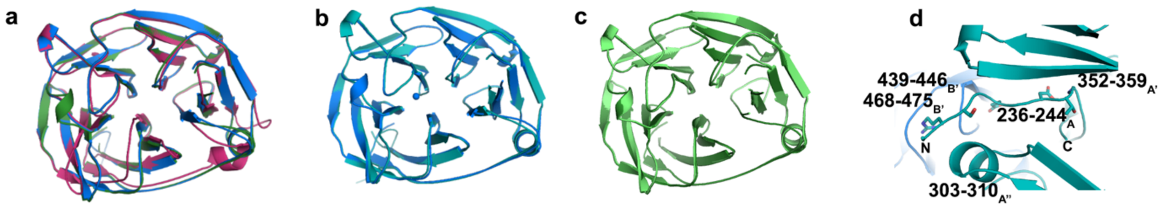

Figure S3 Additional structural details of myoc-OLF$^{N428E/D478S}$ (green), myoc-OLF$^{N428D/D478H}$ (teal) or myoc-OLF$^{N428E/D478K}$ (magenta). (a) Superposition of three structures newly solved in this manuscript. (b) Superposition of both monomers of myoc-OLF$^{N428D/D478H}$ in asymmetric unit (c) Superposition of both monomers of myoc-OLF$^{N428E/D478S}$ in asymmetric unit. (d) Zoom into far-N terminus of myoc-OLF$^{N428D/D478H}$.

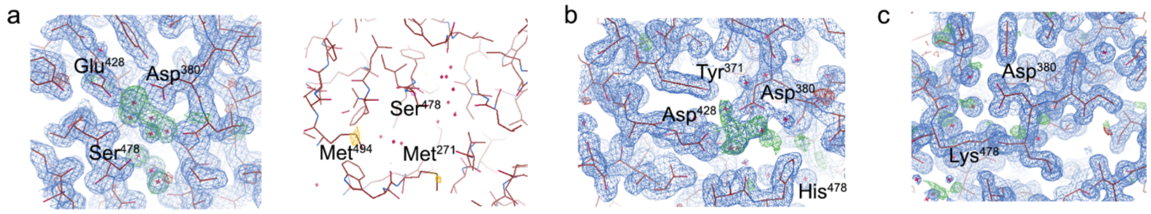

Figure S4 Representative electron density maps. (a) Left, 2Fo-Fc (1 σ, blue mesh) for final model of myoc-OLF$^{N428E/D478S}$ superimposed with Fo-Fc (3σ green mesh) calculated using the final polypeptide chain (central non-covalent metal ions and waters were removed). Right, anomalous difference map (4σ gold mesh) from separate dataset, showing anomalous signal for methionines but not a central Ca^{2+}. (b) Final 2Fo-Fc (1 σ, blue mesh) map for myoc-OLF$^{N428D/D478H}$ (c) Final 2Fo-Fc (1 σ, blue mesh) and Fo-Fc (±3σ green/red mesh) maps for myoc-OLF$^{N428E/D478K}$. View presented is the environs of the central cavity and triads of interest. Maps generated in Coot.

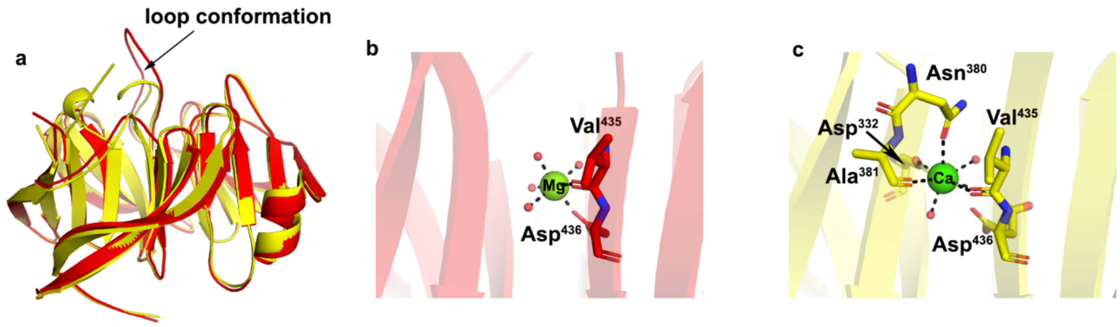

Figure S5 **LPHN3 conformations.** (a) Superposition of PDB code 4RML (red) and 4RMK (yellow) showing altered top loop. (b) Zoom into metal site of 4RML showing coordinated Mg^{2+} ion. (c) Zoom into metal site of 4RMK showing coordinated Ca^{2+}.

Table S1 Primers used in this study.

Mutation	Primer
N428E	Fwd 5’GTAAGCAGTCAGTCGCCGAAGCCTTCATCATCTGTGG
	Rev 5’CCACAGATGATGAAGGCTTCGGCGACTGACTGCTTAC
N428D	Fwd 5’CGTAAGCAGTCAGTCGCCGATGCCTTCATCATCTGTG
	Rev 5’CACAGATGATGAAGGCATCGGCGACTGACTGCTTACG
D478K	Fwd 5’CAGCAGCATGATTAAATACAACCCCCTGG
	Rev 5’CCAGGGGGTTGTATTTAATCATGCTGCTG
D478H	Fwd 5’CAGCAGCATGATTCATTACAACCCCCTGG
	Rev 5’CCAGGGGGTTGTAATGAATCATGCTGCTG
D478S	Fwd 5’CCGCTATAAGTACAGCAGCATGATTAGCTACAACCCCCTG
	Rev 5’CCAGGGGGTTGTAGCTAATCATGCTGCTGTACTTATAGCGG
